# Up, Down, and All Around: Courtship Behavior Deviants and Genetic Divergence in the Reef‐Dwelling Bioluminescent Ostracod, *Photeros annecohenae* (Myodocopida: Cypridinidae)

**DOI:** 10.1002/ece3.72227

**Published:** 2025-10-06

**Authors:** Nicholas J. Reda, Vanessa L. González, Todd Osmundson, Gretchen A. Gerrish

**Affiliations:** ^1^ Biology Department University of Wisconsin La Crosse La Crosse Wisconsin USA; ^2^ Global Genome Initiative Smithsonian National Museum of Natural History Washington DC USA; ^3^ Trout Lake Station—Center for Limnology University of Wisconsin Madison Madison Wisconsin USA

**Keywords:** complex courtship, ddRad, marine ostracod, meiofauna, SNPs, speciation

## Abstract

Species that have complex courtship behaviors are some of the most evolutionarily diverse lineages observed in nature. Divergent, pre‐mating calls are effective in both generating and maintaining reproductive isolation. Complex courtship displays provide numerous traits in which a small change can reinforce or lead to reproductive isolation. Display traits often evolve interactively, multiplicatively increasing the potential phenotype variants. Because many traits can be quantified and used to document variation among species, organisms that use complex courtship behaviors provide model systems for testing the influence of ecology on lineage diversification and trait evolution. Here, we quantify differences in the courtship behavior, morphology, and genetic trait change of male *Photeros annecohenae* over an intermediate range of geographic distances along reef habitats of the Mesoamerican barrier reef of Belize. Differences in bioluminescent ostracod behavior, morphology, and genetics have been documented across large geographic distances (500–1000 km) and at smaller geographic scales (~12 km), but intermediate spatial scales have not previously been evaluated. We found significant differences in observed behavioral, morphological, and genetic traits across isolated populations of *P. annecohenae* resulting from both isolation by distance (IBD) and isolation by barrier (IBB). Furthermore, we describe a newly discovered downward displaying behavioral variant population of *P. annecohenae* nested within the upward displaying populations. The morphological, behavioral, and genetic variability documented for *P. annecohenae* across the 196 km seagrass mosaic of the Mesoamerican reef system offers novel insight toward our understanding of the speciation continuum and the role of complex behavioral courtship in promoting divergence within taxa.

## Graphical abstract

Species that have complex courtship behaviors are some of the most evolutionarily diverse lineages observed in nature. Here, we quantify differences in the courtship behavior, morphology, and genetics of a group of bioluminescent marine ostracod residents of the Mesoamerican barrier reef of Belize. Evidence supports that populations have diverged due to isolation by distance and isolation by barrier and that anomalous behavioral variants can arise preceding or independent of genetic or morphological divergence.

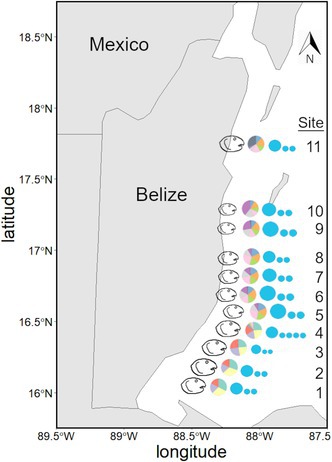

## 1 Introduction

While ecology provides opportunity for diversification, key innovations in behavior can rapidly increase the rates of speciation within groups (Mendelson et al. 2014; Ellis and Oakley 2016; Martin and Mendelson 2016; Janicke et al. 2018; Shaw et al. 2024) and can either initiate and/or maintain reproductive isolation. Behavioral pre‐mating reproductive isolation can drive speciation through the divergent coevolution of male mating signals and female mate preference (Panhuis et al. 2001; Ritchie 2007; Safran et al. 2013). Birds, frogs, and crickets are strong examples of clades that exhibit rapid lineage diversification through key innovations in acoustic mate display (Kaiser et al. 2018; Graham et al. 2018; Blankers et al. 2018). The Hawaiian cricket *Laupala* has radiated across islands due to the tight coevolutionary bond of male signals and female preferences (Blankers et al. 2018). In this case, geographic variation and directional sexual selection of multivariate male acoustic characters together play an important role in patterns of song divergence (Oh and Shaw 2013). The Central American red‐eyed tree frog has strong population‐level differentiation in male acoustic and visual communication, together enhancing local population mate preference (Kaiser et al. 2018). Lineages that use multivariate or complex communication signals are ideal for testing the role of reproductive behaviors as drivers of speciation.

Many terrestrial and freshwater taxa provide models for evaluating speciation mechanisms in time and space, but limited work in marine systems extensively applies these models to explain highly diverse groups. Compared to freshwater systems where organisms must penetrate the water‐air boundary multiple times to disperse, marine systems are vastly connected, and defining connectivity depends on understanding life history and dispersal of target taxa (Palumbi 1994). Many marine flora and fauna rely on large‐scale thermal ocean currents for dispersal and migration. In marine coastal regions, where physical barriers to dispersal are absent, temperature can initiate ecological speciation (Teske et al. 2019). For example, in the Indonesian Spermonde archipelagos, vertebrate ( *Amphiprion ocellaris* ) and invertebrate (*Polycarpa aurata*) species had panmictic population structures on the northern end where there are stronger ocean currents and had significantly restricted gene flow at small geographic scales on the southern end where there are weaker currents and a shallow reef shelf (Timm et al. 2017). However, strong currents, upwelling, and geographic distance may act to restrict gene flow in the New Zealand sea urchin, *Evechinus chloroticus*, where there is strong fine‐scale and broad‐scale population differentiation (Nagel et al. 2015). Small‐scale population genetic analyses are fundamental to understanding the complex, interwoven biological mechanisms (larval duration, larval behavior, isolation by distance, limited dispersal capabilities) that drive genetic divergence (Pascual et al. 2017). The complex, interconnected relationship between abiotic and biotic factors results in a level of spatial genetic differentiation greater than expected (Charrier et al. 2006; Calderón et al. 2007; Palero et al. 2008). Benthic marine organisms in their larval stage can be transported substantial distances and are affected by many of these factors, which play a significant ecological and evolutionary role in adult marine populations and their connectivity. Life history characteristics include adult mobility, breeding or broadcast spawning modes of reproduction, and larval development, motility, and behavior. Abiotic (e.g., geographical and physical) factors include oceanic currents, tidal currents, eddies, and geographic location and history. These abiotic factors, especially geographic barriers, significantly affect dispersal, gene flow, and population connectivity (Treml et al. 2008; Rasmussen 2009).

The Meso‐American barrier reef off the coast of Belize has a suite of small island‐like cayes and large cuts in the forereef that provide an intriguing study environment to assess regional population dynamics of marine coral reef organisms. These geographic structures are capable of restricting gene flow between populations and initiating reproductive isolation. For example, Caribbean reef hamlets are speciating in full sympatry with extensive gene flow across macro‐ and microgeographic scales, where alleles responsible for mate recognition, in vision and pigmentation, are maintained via a long‐scale linkage disequilibrium and driven by both assortative mating and natural selection (Hench et al. 2019). At regional or intermediate spatial scales, there is a lack of information on the connectivity between local and global populations and the eco‐evolutionary forces and that may be responsible for morphological, ecological, and/or genetic character state divergence (Edgar et al. 2017). The non‐uniform ecological fragmentation of the Meso‐American barrier reef may be driving high prezygotic isolation in populations at variable spatial scales.

Bioluminescent ostracods (Myodicopida: Cypridinidae) that use light signals for courtship have a wide geographic distribution in the Caribbean Sea (Morin 2019) and produce one of the most complex luminescent courtship displays described in marine systems (Morin 1986; Rivers and Morin 2008, 2009). Multiple species of ostracods can be observed displaying in the same geographic region, partitioning their mating displays in space and time. When geographically isolated, sister taxa maintain some display characteristics, but the direction, timing of displays, and habitat in which displays take place change (Gerrish and Morin 2016). The dynamic multivariate courtship display traits and spatio‐temporal habitat used for mating creates a landscape for divergence on top of the already heterogeneous ecological opportunity of the reef habitat. At a large scale, surveys throughout the Caribbean Sea (Panama, Belize, Puerto Rico, Roatan, Jamaica) indicate that each collection location has a unique assortment of species that differ in behavioral, morphological, and genetic characters (Ellis et al. 2023). On the small scale, *Photeros annecohenae* (a grass‐bed dwelling species) (Torres and Morin 2007) shows strong population genetic structure across a 12 km range (Gerrish 2008). While sister taxa show clear genetic differentiation at 500–1000 km scales (Ellis et al. 2023), and population genetic structure can be identified at 0–12 km scales (Gerrish 2008), population divergence resulting in speciation is likely happening somewhere between small and large spatial scales. The research presented here aims to (1) identify the geographic distance at which morphological, behavioral, and genetic trait change is occurring, (2) assess whether trait change is associated with barriers to gene flow or dispersal limitation, and (3) to discuss how sympatric (behavioral isolation) and allopatric (geographic isolation) barriers of diversification apply to populations of *P. annecohenae*.

## 2 Materials and Methods

### 2.1 Site Selection

**FIGURE 1:**
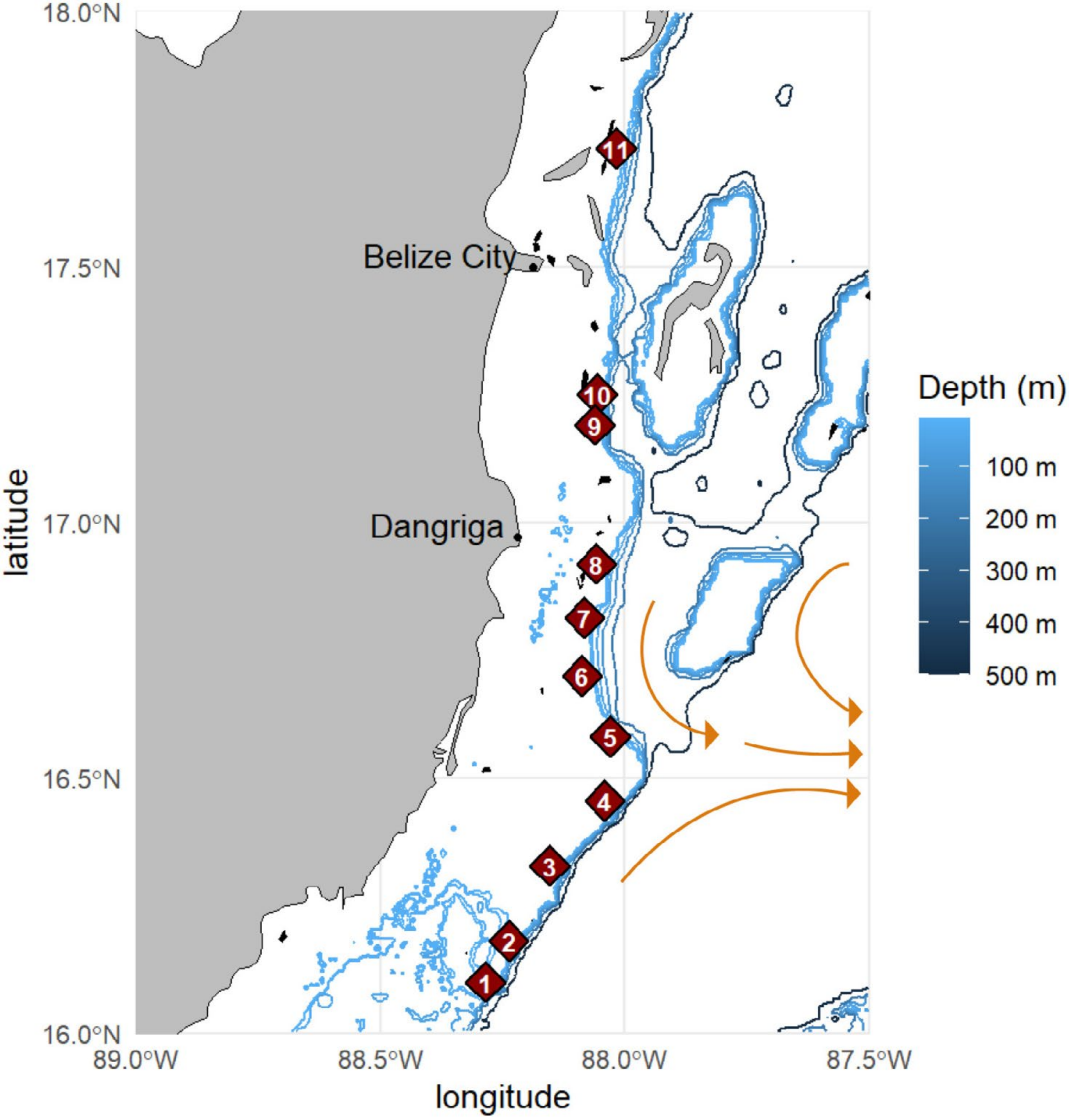
Sampling locations (numbered diamonds correspond to sites listed in Table 1) of *P. annecohenae* along the Meso‐American barrier reef adjacent to Belize, CA. Arrows represent the dominant deep water current directions coming offshore and circulating through the Cayman Trench (modified from Heyman and Kjerfve 1999).

Sample sites were selected within a 196 km range of the Meso‐American barrier reef of Belize (Figure 1). Approximate locations were selected based on nautical depth contour maps in locations where seagrass habitats were visible on Google Earth at distances of 15–20 km apart. Upon arrival at locations, sites were selected to allow consistent depth (5–20 ft) and prominent seagrass, *Thalassia testudinum*, the preferred habitat conditions of *P. annecohenae*. Distance between all sites ranged from 9 to 184 km (Figure 1), and neighboring sites ranged from 9 to 17 km apart. Multiple sampling locations were planned between sites 10 and 11, but no luminescent displays were observed at these locations while diving at night in the appropriate habitats. Video and specimens were collected from sites 1–11 during a single lunar cycle from May 1 to May 10, 2018 (Table 1).

**TABLE 1:** Site descriptions of locations where collection of *P. annecohenae* took place on the Meso‐American barrier reef of Belize, CA.

Site	Nearby island	Latitude	Longitude	Seagrass density	Seagrass height (cm)	Depth (m)	Sampling date (dd/mm/yr)
1	Ragged	16.10001	−88.286	Medium	25	4	10/05/2018
2	Tom Owens	16.18029	−88.2376	Medium	25	5	10/05/2018
3	Ranguana	16.32794	−88.154	High	30	6–7	03/05/2018
4	Three Queens	16.45488	−88.0414	Medium high	30	7	03/05/2018
5	Gladdens	16.58051	−88.0302	Low	25–30	4	02/05/2018
6	Carrie Bow	16.69883	−88.0873	Low	25–30	4	02/05/2018
7	South Water	16.81255	−88.0827	Medium	30	2	01/05/2018
8	Tobacco	16.91851	−88.0582	High	20	4	01/05/2018
9	Gator Caye	17.19043	−88.062	Medium high	20–25	2–4	08/05/2018
10	Middle Long	17.25076	−88.0563	Medium high	25	3	05/05/2018
11	Caye Caulker	17.73154	−88.0173	Medium high	25–30	3	07/05/2018

*Note:* Site numbers are associated with nearby islands.

### 2.2 Animal Collection and Identification

We collected signaling male *P. annecohenae* during their bioluminescent signals and courtship displays by sweeping a handnet (125 μm) in the direction of the light display (Torres and Morin 2007). At each site, we swept 60 *P. annecohenae* displays following the protocol of Gerrish and Morin (2016). Live *P. annecohenae* individuals were sorted by their relative length:height ratio and keel shape (*N* = 30) (Torres and Morin 2007). Individuals from each population were measured on a Nikon SMZ‐800 microscope with an eyepiece micrometer at 50× magnification. Length and height measurements were taken from 30 individuals, with keel and eye measurements taken from 15 of the same individuals (Gerrish and Morin 2016; Morin and Cohen 2017). The 30 measured and recorded individuals were then placed in 1.5 mL centrifuge tubes and preserved with 100% ethanol to serve as vouchers and for additional morphological diagnoses. An additional 30 individuals were preserved in 1.5 mL centrifuge tubes with 100% ethanol for genetic analyses.

### 2.3 Behavioral Analysis

Courtship displays of multiple individuals from each population were recorded using a Sony A7S low‐light camera with a 25 mm Canon lens, recording in 4K on a Shogun recorder. The camera was housed in a Nauticam custom underwater housing with a dome port (Settings: 30 FPS; blue light; 124,000–260,000 iso; manual focus). Displays were included in the analysis if clean first and last pulses were captured and in focus. Ten displays from each site were analyzed for the number of pulses, pulse duration, and inter‐pulse interval. Display independence is highly likely given that hundreds of males are simultaneously displaying within the video arena and because we often move 1–2 m between capturing a successful video of a full display, but observation independence cannot be guaranteed. Pulse duration was quantified as time (seconds) from the initial observed luminescence to no luminescence observed. Inter‐pulse interval was quantified from the beginning of one pulse to the start of the following pulse.

### 2.4 Molecular Techniques and Analyses

Male *P. annecohenae*, between 15 and 16 individuals from each of the 11 sampled sites, were dissected and stomach contents were removed to reduce DNA contamination. Genomic DNA (gDNA) was extracted with a proteinase K treatment following the Qiagen protocol for animal tissue (with one modification: the first elute was 100 μL instead of 200 μL). Extracted gDNA was quantified using Qubit dsDNA HS assay kits (Invitrogen) and samples were only kept in the analysis if they yielded 3.5 ng/μl and higher concentrations of gDNA. Hind‐III (New England Biolabs) was used to test digestive quality of DNA and assayed on a 1% agarose gel (i.e., size and ability to ligate [> 95%]). Two 96‐well plates with a total of 190 gDNA extracts (15–16 upward displaying individuals × 11 sites and 16 downward displaying individuals from site 9) were prepared and sent to the University of Wisconsin–Madison Biotechnology Center (University of Wisconsin–Madison, Madison, Wisconsin) (UWBC). Double‐digest RAD‐sequencing libraries were prepared by UWBC, and enzymes PstI and MspI were selected to use for the digest based on optimization. Two libraries were produced containing 95 individuals each, barcoded with a unique 4–9 nucleotide sequence. Single‐read, 100‐bp target length sequencing on Illumina HiSeq2500 platform was conducted at the UWBC. All raw demultiplexed sequences used in downstream analysis have been deposited on the NCBI sequence read archive (SRA) database under BioProject ID: PRJNA1230213.

Libraries were demultiplexed using the *process radtags* program in STACKS (Catchen et al. 2013). Polymorphic SNPs were identified on reads truncated to 100 bp and filtered for overall quality. RAD loci were allowed a maximum of three nucleotide mismatches (*M* = 3), which were identified as an optimum threshold based on the method developed by Catchen et al. (2013). We selected a minimum stack depth of three (*m* = 3) among reads with variable sequences (*ustacks* module in STACKS, default parameters selected). Reads were then aligned de novo with each other to create a catalog of putative RAD tags (*cstacks* module in STACKS, default parameters selected). Samples with a minimum of 1.5 million reads passing the filter were used in all downstream analyses in STACKS (132 individuals). In the *populations* module of STACKS, we retained SNPs in 70%, 75%, 80%, and 85% of the individuals and sampling sites (*r*). Amplification for some individuals was unsuccessful, and some did not reach data quality thresholds to be included, leaving sites represented by between 8 and 16 individuals in the final genetic analyses. Homeologs were excluded by removing markers showing heterozygosity > 0.70 within samples. Polymorphisms with a minor allele frequency (MAF) > 0.05 on average across sampling sites were kept to avoid bias in baseline differentiation and eliminate any sequencing error from the SNP data set. Sequences were also assembled using ipyrad v. 0.6.8 (Eaton 2014). Samples with a minimum of 1 million reads passing the filter were used in all downstream analyses in ipyrad (152 individuals). In ipyrad, reads were clustered within and between samples at 85% identity with 50% missing data; all other parameters were set at default. In both STACKS and ipyrad pipelines, parameters were optimized for clustering (reducing under/over clustering) and matrix density (allowable missing data). VCF file formats were generated from both STACKS and ipyrad pipelines to be used for statistical analyses in R v. 3.0.6 (R Core Team 2019). To test for population genetic structure, PCA and DAPC analyses were run using the adegenet package (Jombart and Ahmed 2011; Grünwald and Goss 2011; Jombart et al. 2010) in R v. 3.6.0. To test for isolation by distance, Nei's genetic distances between sites were calculated in the genlight package (R v. 3.6.0) and compared with geographic distances between sites using a Mantel test based on Pearson's correlation coefficient.

Differences in pulse number per bioluminescent display among sites across sample sites were evaluated using ANOVA in R v. 3.6.0. To test for site differences in pulse duration and interpulse interval, we conducted separate repeated‐measures linear mixed model in R v. 3.6.0. Ten individual displays were recorded at each site and treated as random samples nested within the site (with the exception of site 10, where low display density only allowed for 5 recordings). It is possible that we occasionally captured displays from the same individual, but it is highly unlikely given that most sites had hundreds of displays occurring simultaneously, and we often moved 1–2 m between successful recordings. We categorized the first 5 pulses as a fixed, sequential factors since each subsequent pulse duration is likely dependent on the prior. Interpulse intervals between the first 6 pulses were similarly treated as fixed, sequential factors and tested in a separate repeated‐measures model. For both analyses, Tukey's post hoc analysis was used to determine which sites differed significantly (*p* < 0.05).

## 3 Results

### 3.1 Size

**FIGURE 2:**
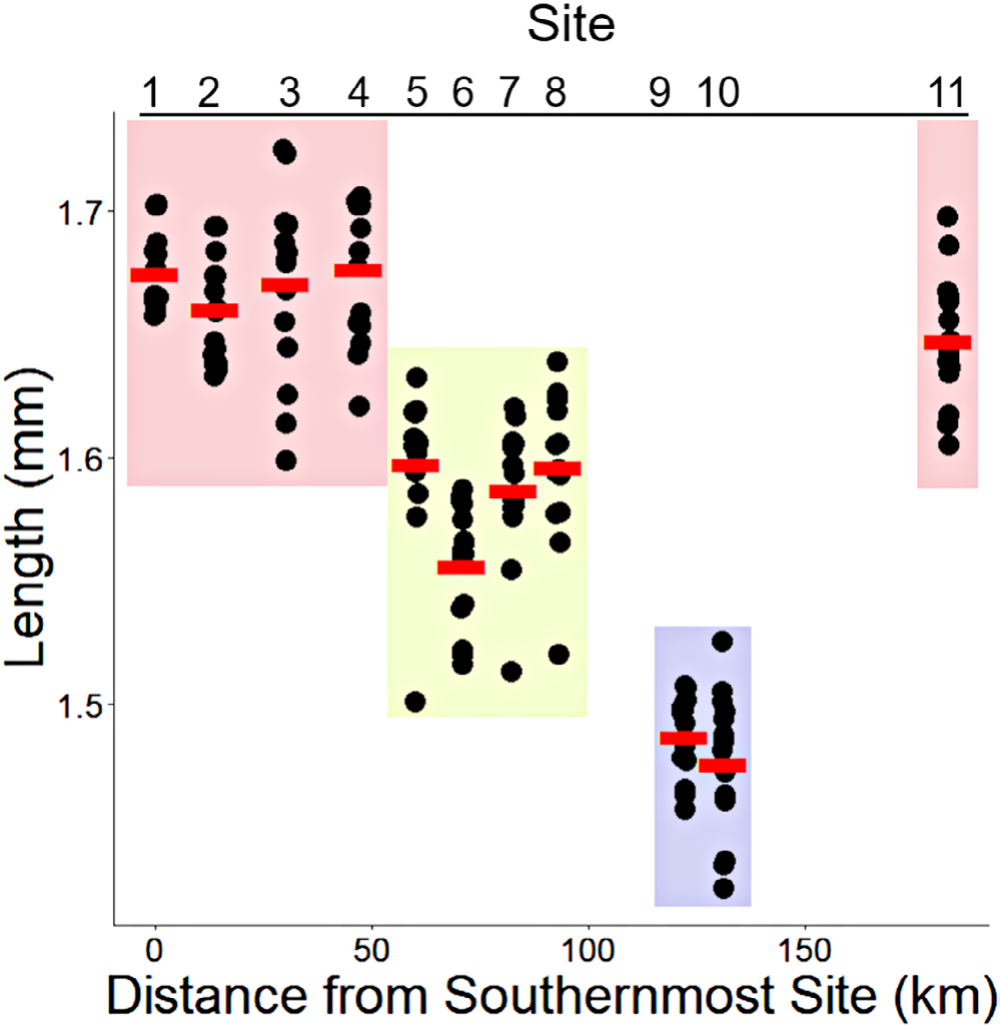
Mean length (mm) compared across sampled sites. Statistically similar sites (based on one‐way ANOVA and post hoc Tukey analyses) are highlighted with similarly colored boxes.

Ostracod size is presented as length, the distance from the outer edge of the keel to the tip of the rostrum, as illustrated in Morin and Cohen (2017, [figure 10]). Length was highly correlated with height for all measured individuals (Pearson *r* = 0.93, *p* < 0.001), so we used length comparisons to illustrate size differences across sites. Mean body length significantly (ANOVA, *p* < 0.0001) decreased from south to north with larger individuals in the south (sites 1–4), medium size in the central sites (5–8), and smallest at sites 9 and 10. The exception was site 11 where *P. annecohenae* were similar in size to individuals from the southern sites (Figure 2). Keel and eye size varied minimally across sites and only site 4 had significantly larger mean eye size than some other sites.

### 3.2 Behavior

**FIGURE 3:**
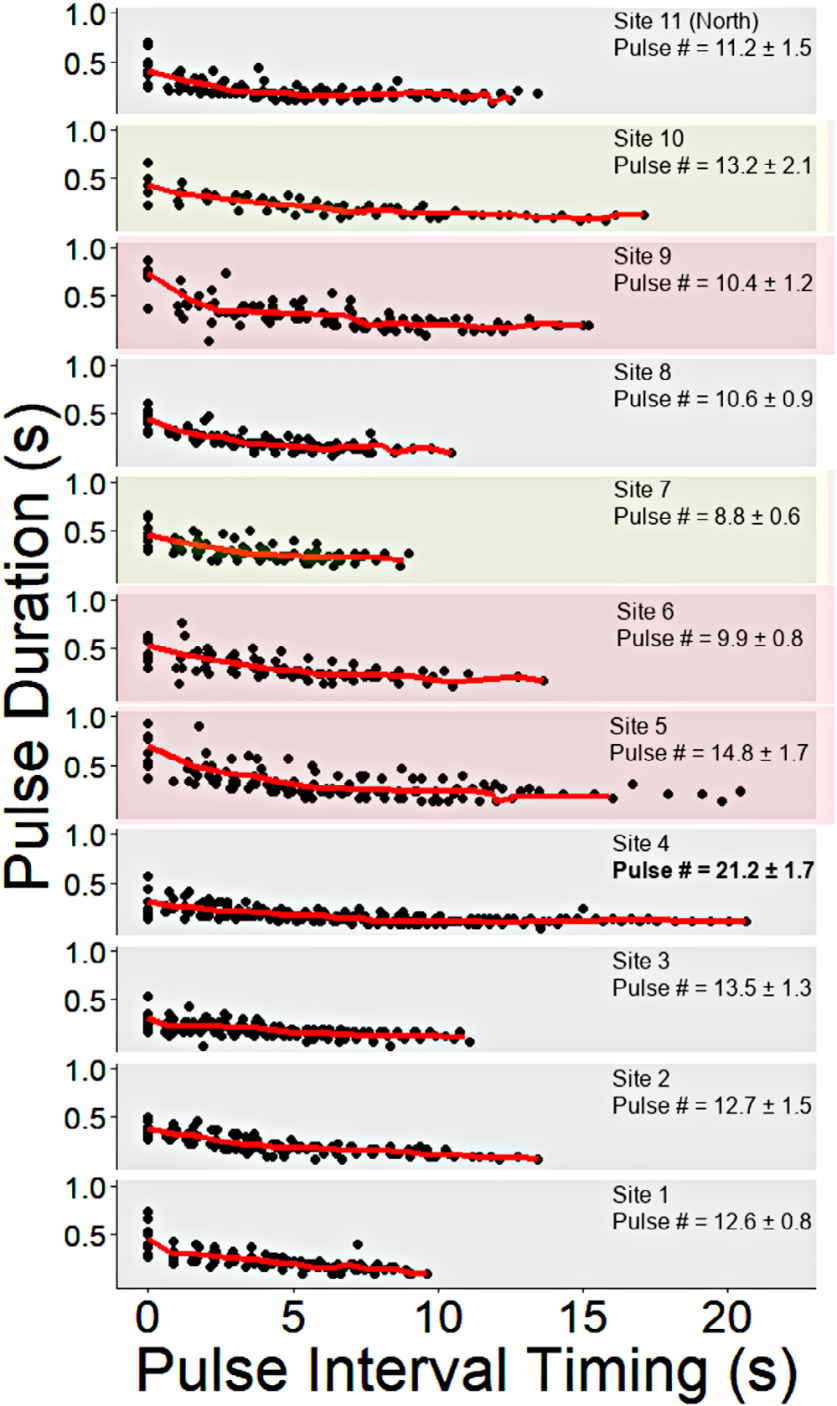
Courtship light display characteristics compared across sites; (*N* = 10) for all except site 10 (*N* = 5). Black dots represent timings for each individual and red lines represent mean pulse duration and pulse interval for each pulse of the luminescent display at each site. Statistically significant differences are represented in pulse number by bold text at site 4. Red boxes represent sites (5, 6, and 9) that have longer pulse duration and interpulse intervals. Yellow boxes represent sites that have longer pulse duration (site 7) or longer interpulse intervals (site 10).

At all sites, *P. annecohenae* were observed conducting flashing upward displays comparable to those originally described in Torres and Morin (2007) and further detailed in Rivers and Morin (2008). The displays consisted of 2–3 slightly longer duration initial pulses as part of the initiation phase and then trilled into a series of shorter pulses before stopping. Site 4 had significantly more pulses as part of their displays than all other sites (one way ANOVA, *p*_10_ < 0.05, Figure 3). Pulse durations were significantly longer at sites 5 and 9, moderately long at sites 6 and 7, and shorter at all other sites (based on a repeated measure linear mixed model for the duration of the first 5 display pulses, Tukey *p* < 0.05, Figure 3). Interpulse intervals were significantly longer at sites 5, 6, 9, and 10; moderately long at sites 7, 8, and 11; shorter at sites 1, 2, and 4; and shortest at site 3 (based on a repeated measure linear mixed model for the duration of the intervals between the first 6 display pulses, Tukey *p* < 0.05, Figure 3).

### 3.3 Genetics

In total, we obtained an average of 2.75 ± 0.99 (SD; standard deviation) million reads per individual, 2.74 ± 0.98 million reads retained after quality filtering. Within STACKs, after applying a < 50% missing data filter, removing low coverage samples (samples with < 1.5 million reads passing filter), and selecting for 70%, 75%, 80%, and 85% of the individuals and sampling sites (*r*), we recovered 99,060, 71,839, 51,871, and 22,340 SNPs, respectively. In ipyrad, after applying a < 50% missing data filter and removing low coverage samples (samples with less than 1 million reads passing filter), we generated a matrix containing 994 SNPs representing 152 individuals.

**FIGURE 4:**
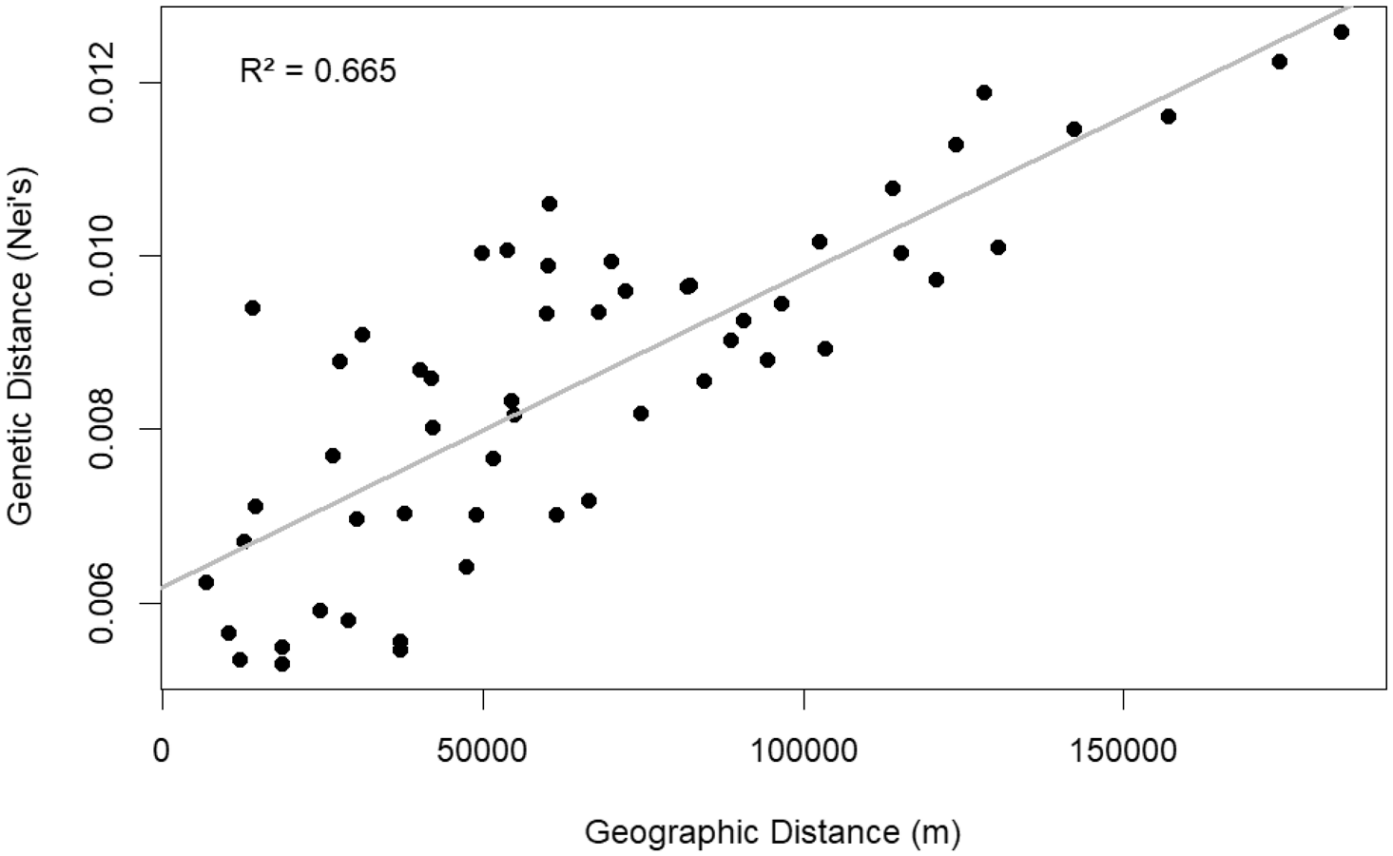
Isolation by distance analysis comparing the geographic distance and calculated Nei's genetic distance between the 11 sites sampled along the Belize barrier reef.

Geographic distance strongly correlated with Nei's genetic distance (Figure 4, *R* ^2^ = 0.665), indicating significant overall isolation by distance (*p* < 0.001, Mantel test on Pearson Correlation coefficient). And while the linear relationship is significant, some close proximity populations have higher and lower genetic distances than expected, indicating that distance may not be the only isolating factor.

**FIGURE 5:**
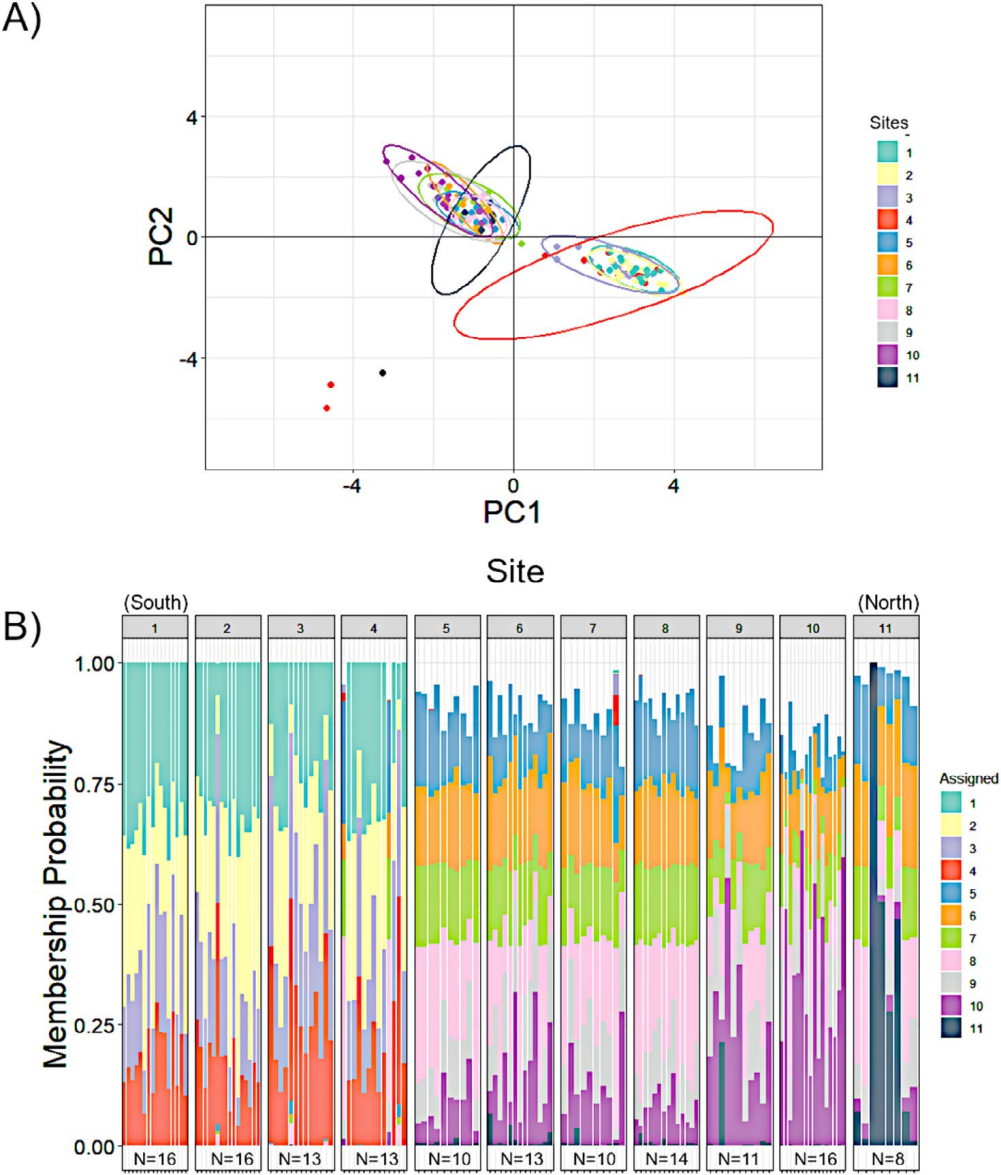
*Photeros annecohenae* population assignment probabilities from (A) principal components analysis (PCA); color coded by site with ellipses representing 95% of all individuals from each site and (B) discriminant analysis of principal components (DAPC) of SNPs for individuals from each site; each vertical bar represents an individual, where the length of the colored segments are proportional to the estimated membership probabilities grouped by site. Black boxes separate each of the 11 sites, and the total number of individuals (N) represented are shown at the bottom of each site segment.

DAPC and PCA analyses using the SNP data generated in STACKS and ipyrad showed nearly identical results in assigning populations, with admixture present among sites in all populations. Using the results generated in ipyrad, individual population assignment (DAPC) and principal component analyses (PCA) support that the southern (sites 1–4) and northern populations (sites 5–11) of *P. annecohenae* are genetically diverging (DAPC presented in Figure 5). There is a well‐defined southern population that includes all individuals from sites 1–4 (Figure 5A). One individual from site 4 showed a somewhat northern signature, but there is a strong genetic boundary between sites 4 and 5. The northern population shows more variability among individual assignment probabilities and suggests three sub‐populations: (a) sites 5–8; (b) sites 9 and 10; and (c) site 11 (Figure 5B).

### 3.4 Summary

*Photeros annecohenae* collected from the four southernmost sites were genetically and morphologically similar, and the courtship displays recorded at each site had shorter pulse durations and interpulse intervals. Site 3 displays did have significantly shorter interpulse intervals, and displays at site 4 had many more pulses per display than any of the other southern sites (nested ANOVA, Tukey post hoc *p* < 0.05). We also observed that at these southern sites the signaling ostracod would commonly “skip” a pulse during the last half of their display; after 8–10 pulses there would be a longer interpulse interval, and then 4–5 more pulses would occur. This “skip” behavior was not observed in the northern sites.

**FIGURE 6:**
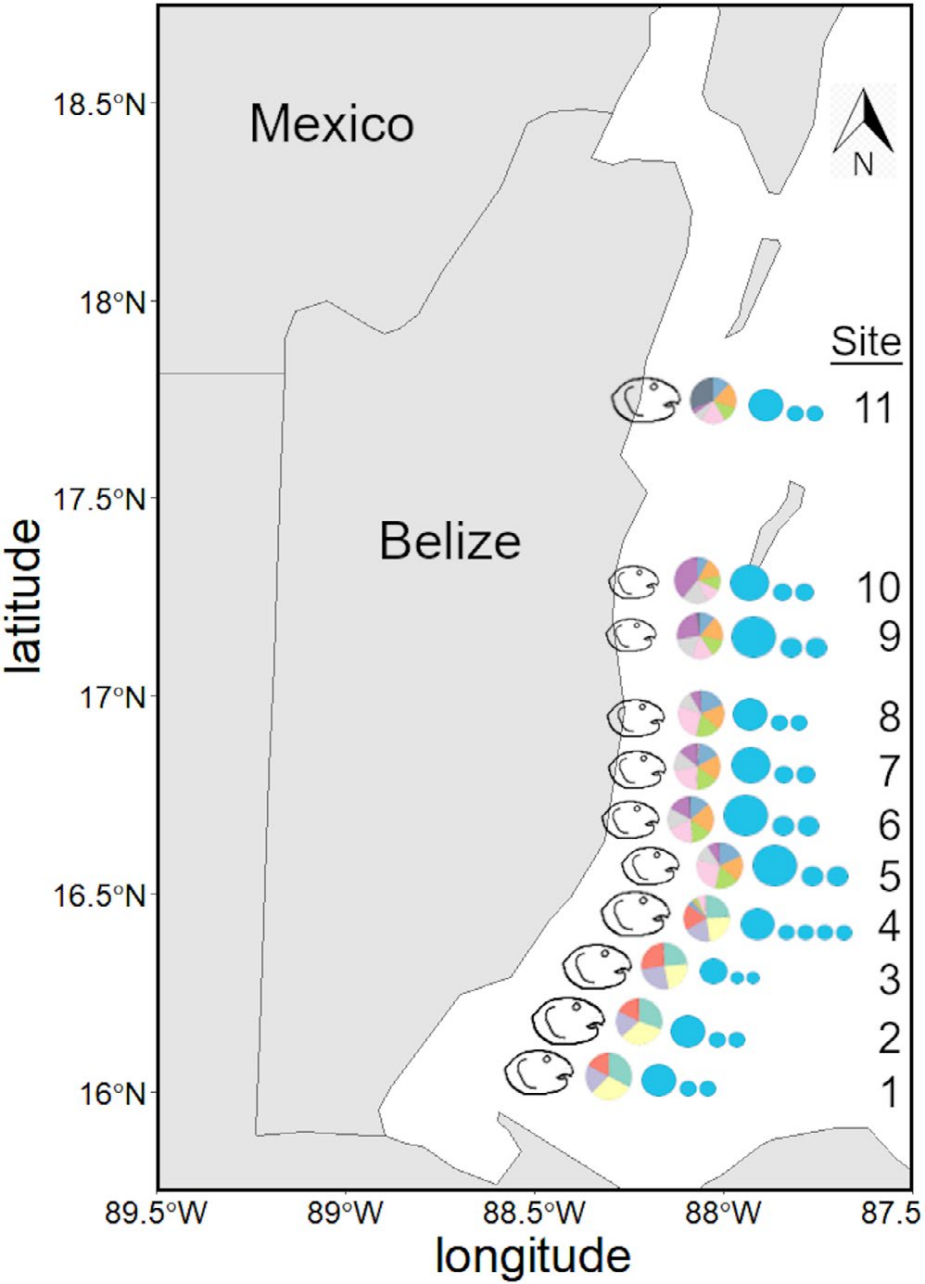
Mapped summary of population assignment, courtship signal, and morphological differences across sites. Pie charts represent population assignment (color legend shown in Figure 4), dot symbols represent courtship traits (larger circles = longer duration pulses and/or interpulse durations; more dots = displays with more pulses), and *P. annecohenae* images are scaled to represent sites with morphologically small, medium, or large sizes.

*Photeros annecohenae* from sites 5–8 were also genetically and morphologically similar. Courtship displays at sites 5 and 6 had long mean pulse and mean interpulse durations, and site 7 had long pulse durations. Displays at site 8 looked more like those observed at the southern sites 1–4. *Photeros annecohenae* from sites 9 and 10 was significantly smaller in size than those from all others. The genetic population assignment for sites 9 and 10 shows admixture with individuals from sites 5–8 but a slightly different genetic structure. Courtship displays at site 9 looked like those at sites 5 and 6 but with longer pulse durations and interpulse intervals. Site 10 displays also had longer interpulse intervals, but pulse durations were not as long as those at site 9. Site 11 is anomalous in that the morphology and display traits are like those observed in the southern sites 1–4, and the genetics have more admixture with sites 5–10 with some variants. *Photeros annecohenae* at site 11 has a large body size, and displays have short pulse durations and interpulse intervals (Figure 6).

### 3.5 Up Versus Down at Site 9

**FIGURE 7:**
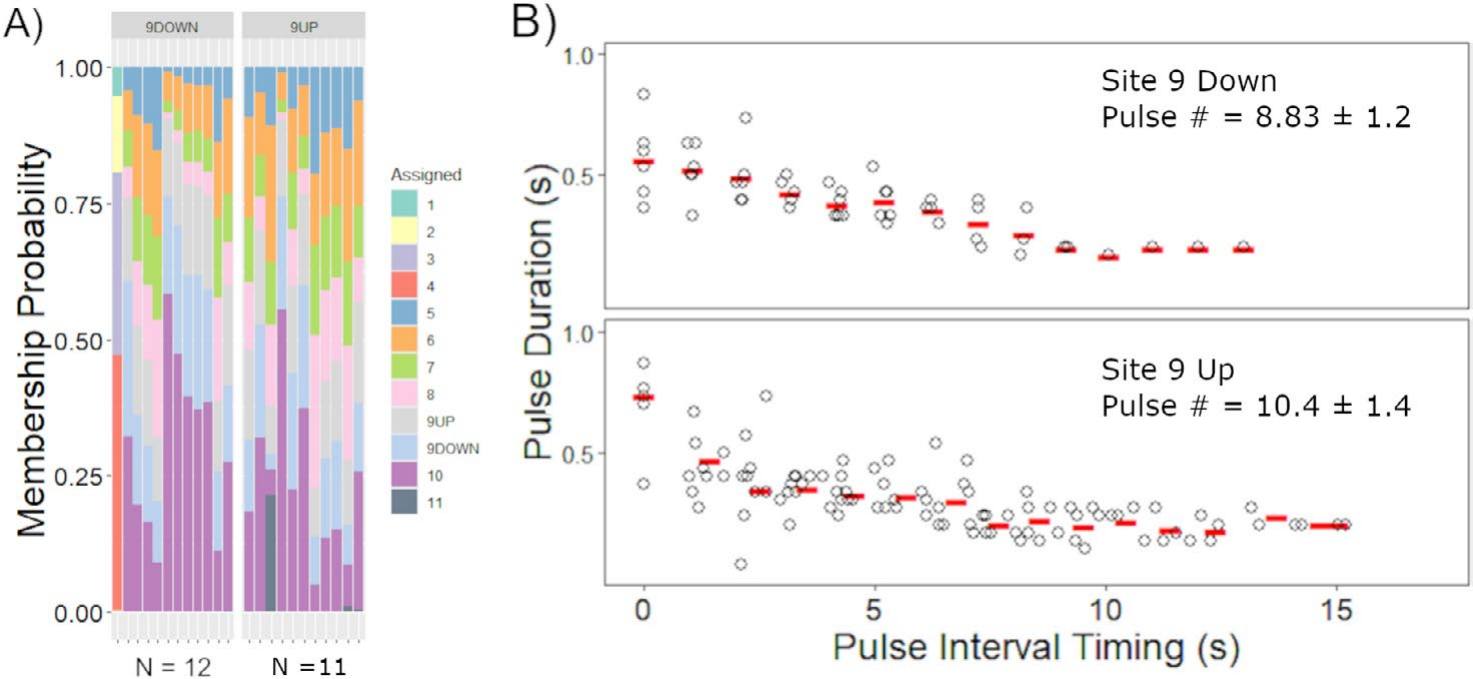
*Photeros annecohenae* (A) population assignment probabilities and (B) courtship light display characteristics compared between upward and downward displaying male ostracods from site 9.

Across all sites, *P. annecohenae* males performed upward flashing luminescent courtship displays that only varied slightly from those described for the species (Torres and Morin 2007). But, at site 9, approximately 70% of the males swam downward while emitting the flashing pulses of light completely inverse to the commonly observed upward displays (Figure 7). Upward and downward displaying individuals performed their signaling behaviors immediately adjacent to each other along the back reef sloping seagrass bed. The contrast was striking compared to all other sites where only upward displays were observed. Downward displaying males started their displays slightly higher in the water column ~20 cm above the top of the seagrass while upward displaying males started within the seagrass and finished their displays 20–40 cm above the seagrass. Upward displaying individuals had a slightly longer first pulse and interpulse interval between pulses one and two, but otherwise, the pulse duration and number of pulses were mostly similar. There were no clear genetic differences (Figure 7) or morphological differences between upward and downward displaying males.

## 4 Discussion

While morphological or behavioral changes can act independently to generate reproductive isolation, it is more common that both are observed in a divergence event (Warner and Schultz 1992; Momigliano et al. 2017). We observe clear differences in phenotypes, both behavioral and morphological, for *P. annecohenae* between sites 4 and 5. Pulse duration, inter‐pulse interval, and body size all significantly change. Genetics supports that these phenotypic changes take place in the same region where we observe significant genetic differences (Figure 6). There is an overall pattern of genetic isolation by distance (IBD) among sampled sites (Figure 4), but we suggest that isolation by barrier (IBB) has had a strong influence on the break identified between sites 4 and 5 (Figure 5). Site 4 is located near Gladden Island, and just north of Gladden's Spit, which is well known for its large grouper (Epinephelidae) and snapper (Lutjanidae) spawning events that draw in whale sharks each spring (Heyman and Kjerfve 2008). Flow dynamics at Gladden's Spit have been studied extensively to document why fish aggregate at this location where converging currents push water offshore (Figure 1). Flow dynamics within the Caribbean vary seasonally but commonly form eddies and anticyclonic strong southern surface currents in association with an underwater cape present at this location (Thattai et al. 2004; Karnauskas et al. 2011). Seasonal currents help carry embryonic fish into good habitats for development (Heyman and Kobara 2012; Karnauskas et al. 2011). High flow conditions are known to limit ostracod activity (Gerrish et al. 2009) and dispersal. Prominent flow direction could explain the presence of a genetic barrier between sites 4 and 5. The morphological and behavioral differences between the northern and southern ostracod populations may have arisen due to limited gene flow at this boundary. IBB is a known driver of genetic divergence. Complex courtship behaviors and lock and key morphology may act to accelerate reproductive isolation when extrinsic barriers exist. Cross‐boundary mate recognition studies, testing whether behavior and morphology are reinforcing reproductive isolation or to identify genetic barrier loci of large effect (Ravinet et al. 2017) between northern and southern populations of ostracods, are needed along this boundary.

In species that have complex courtship behaviors, selection can act on multiple different behavioral character traits to result in reproductive isolation. Taxa that utilize sexual selection, specifically complex courtship displays, have some of the highest diversification rates (Ritchie 2007; Ellis and Oakley 2016). The courtship displays of luminescent ostracods are highly complex and often serve as the first feature by which researchers identify different luminescent ostracod species in the field (Morin 1986; Cohen and Morin 2003; Rivers and Morin 2008). Over a single reef habitat, luminescent courtship displays of male cypridinid ostracods from up to eight species can be simultaneously observed. Each species displays over a specific microhabitat, and each species initiates its displays at slightly offset times (Gerrish and Morin 2016). In addition to differing in direction, species vary in the location, timing, pulse durations, interpulse intervals, kinetics, and coloration of their courtship displays (Hensley et al. 2019). Even though there are so many ways displays can vary, Rivers and Morin (2008) reported that *P. annecohenae* pulse and interpulse characteristics are highly conserved among individuals collected from the grassbeds surrounding South Water Caye (Site 7 in our study). We observe differences of up to 50% in inter‐pulse interval and up to 40% in pulse duration between southern and northern populations of *P. annecohenae* (Figure 3). These are comparable to interspecific differences in display traits observed between *P. annecohenae* and *P. morini* (Torres and Cohen 2005; Torres and Morin 2007; Gerrish and Morin 2016).

Behavioral anomalies can lead to and reinforce reproductive isolation. If reproductive isolation persists, subsequent genetic divergence is likely. Anomalous behaviors are common in complex courtship and mating systems; however, the success of an anomalous behavior requires a level of flexibility in the receiver. And while some behaviors can support species or population isolation, behavioral learning in complex behaviors can also proliferate quickly, generating variable phenotypes with limited genetic or selective consequence (Dion et al. 2019). Published reports of the occurrence of adjacent upward and downward displays exist for *Vargula kuna* (Morin and Cohen 1988). The up vs. down directionality of displays observed at site 9 for *P. annecohenae* represents a second documented occurrence of directional variants and allowed us to test for underlying genetic divergence between the two phenotypes. The two behavioral phenotypes were observed immediately adjacent to each other, initiating displays at the same height and swimming in opposite directions. At site 9, almost 70% of the displays were downward. The large proportion of downward displaying males at these sites supports that there are females receptive to the deviant male signal allowing it to persist. Future research is needed to test whether females at this site respond to both upward and downward displays and/or whether females at these locations have greater flexibility in their display recognition than in other sites where displays are only upward. Genetic isolation was not evident based on the SNP methods applied in this study (Figure 7), but additional testing could aim to investigate the learned vs. genetic basis for the upward vs. downward phenotypes. Fine‐scale morphological analyses are also warranted since the cause of directional variation in displays is unknown.

Direction is a common differentiating trait between luminescent ostracod species that share the water column above coral reefs to conduct their nightly courtship displays. The sister taxa to *P. annecohenae* in Belize, *P. morini* , conducts a flashing downward display. And, the closely related long‐pulsing species described in Gerrish and Morin 2016, nominally named SVU and SVD, also travel in opposing upward and downward directions. It has been suggested that direction is a reinforcement trait that allows for the co‐existence of related species (Gerrish and Morin 2016). Our finding of opposing directionality at site 9 suggests that display direction variants may arise and establish within a population. If shown to cause genetic divergence and subsequently reinforce reproductive isolation, display direction changes would be categorized as intrinsic behavioral mechanisms of speciation. Further inquiry into female preference, genetic identity, and the recurrence of directional anomalies across lineages are warranted.

Besides differences in standard display characters, pulse duration, and inter‐pulse interval, numerous observations in the field and during video footage analysis showed southern males seemingly “skip” a pulse in the latter portion of their sequence of pulses. These gaps occurred when the distance and inter‐pulse interval (duration/time) between the two pulses were nearly twice as long as all other pulses (except for initiation and trill phase). We hypothesize that the “skipped” pulse could reduce “sneakers”, silent male ostracods that have been documented to trail displaying males attempting to intercept incoming females (Rivers and Morin 2009). The cost of skipping a pulse may be that the females also perceive that the male has terminated their display train. “Skipped” pulses were not observed in any northern sample sites.

While behavior plays an important role, divergent morphology can also act to reinforce reproductive isolation. For decades, taxonomy was limited to morphological characters for taxonomic descriptions. To date, morphology is still a useful tool to identify species‐specific characters when paired with the evaluation of genetic and ecological traits. Barriers to reproduction occur when differences in morphology restrict copulation and the formation of zygotes. This concept comes from the lock and key hypothesis, where the structural differences in genitalia prevent the hybridization of species (Dufour 1844). Lock and key reproductive isolation functions in two ways, which are not mutually exclusive: (1) structural differences result in mechanical incompatibilities that reduce successful copulation (Eberhard 1992), and (2) differences in genital characters are recognized and one or both sexes produce a behavioral or physiological response to reduce reproductive fitness or eliminate mating attempts (De Wilde 1964). In some cases, male genitalia are more variable and evolve more rapidly than non‐genitalia morphological traits as a result of sexual selection (Arnqvist 1997; Hosken and Stockley 2004; Eberhard 2010; Klaczko et al. 2015). Our data show that there is a ~25% difference in length and height between the largest individuals of the southern population and the smallest individuals of the northern population. The difference between these two populations is again comparable to the differences observed between the Belize sister taxa, *P. annecohenae* and *Photeros morini* (Torres and Cohen 2005; Torres and Morin 2007; Cohen and Morin 2010; Morin 2019). In addition to the large difference in overall size, there may also be fine morphological differences between the two populations that act to reinforce reproductive isolation. Species within the genus *Photeros* have highly variable copulatory organs across species (Cohen and Morin 2010). And, while not measured in this study, size differences and copulatory organ structures may play a role in mechanical reproductive isolation among the populations studied and luminescent species in general (Cohen and Morin 1990).

Capturing speciation in action and delineating new cryptic species is one of the most challenging aspects of evolutionary biology. Speciation patterns and processes for some model organisms, *Drosophila*, *Caenorhabditis elegans* , *Danio rerio* , *Mus musculus* , African cichlid fish, and three‐spine sticklebacks, have been relatively well documented, but testing speciation in non‐model systems has remained difficult because of the breadth of required understanding. Next‐generation sequencing (NGS) techniques are revolutionizing our ability to study divergence in non‐model organisms. In the reef‐building coral *Acropora*, single‐nucleotide polymorphisms were mapped to a genome to parse out phylogenetic relationships and build a foundation for resolving regions of the genome that influence spawning time (Porto‐Hannes et al. 2015; Rosser et al. 2017). NGS is also providing small non‐model organisms, for which there is no reference genome, the capability to identify population‐level genetic variation (Krehenwinkel et al. 2018; Wells and Dale 2018). Within luminescent ostracods, Hensley et al. (2021) documented episodic diversifying selection across genera using synonymous and nonsynonymous substitution comparisons for the c‐luciferase gene, a gene hypothesized to be related to diversification of the luminescent ostracod clade. With the recent expansion and increased applications for NGS techniques, our understanding of ecological and evolutionary processes has improved. As with the application of any new methodology, there is still skepticism in the biological inferences being made (Vijay et al. 2012; Chaisson et al. 2015). Whether studies use reference‐based or *de novo* approaches, the use of multiple downstream pipelines ensures a robust outlook on the population genetic and demographic inferences (Shafer et al. 2017). In this study, we applied two pipelines, iPyrad and STACKS, to our ddRAD sequencing data to evaluate population structure along a ~200 km range for a non‐model species *P. annecohenae*. Both pipelines confirm that *P. annecohenae* is genetically diverging into two lineages and that this is happening where there is an overall pattern of isolation by distance.

NGS tools can provide insights at multiple scales of genetic divergence using different analyses on a single data set. Whether identifying barriers to reproductive isolation through whole‐genome scans or population structure, inferences made on the scale of geographic and genetic divergence are now becoming more accessible to non‐model organisms. With increased use of reduced‐representation techniques, the framework for understanding the ecological and evolutionary mechanisms of speciation based on these types of data is improving. Ravinet et al. (2017) proposed a road map for elucidating genomic landscapes of species in six steps: (1) know the study system by understanding the ecology, reproductive biology, life history strategies, and geographic distribution; (2) establish the extent of gene flow and understand the demographic history by sampling a study system where divergent populations or species meet; (3) capture the best possible picture of the genomic landscape through NGS *de novo* or whole‐genome assembly; (4) measure genomic factors that contribute to landscape differentiation; (5) reliably identify potential signatures of divergent selection or candidate barrier loci, while taking modifying factors into account; (6) use evidence independent from genomic data by directly testing for signatures of selection on a given locus or genetically map linkage between genotype and phenotype. Our study captured steps 1–3 and provides an opportunity to identify potential morphological and behavioral barrier loci under divergent selection in luminescent ostracods of the Caribbean Sea.

## 5 Conclusion

Our findings support early suggestions that numerous cryptic species exist throughout the ranges of luminescent marine ostracod species. Divergence increases with and can be amplified by distance, flow corridors, or unfit habitats. Courtship display and morphology changes aligned with genetic differences across the geographic region in *P. annecohenae*, *suggesting* differences observed in behavior and morphology are great enough to reinforce genetic isolation. Behavioral variants within sites were also observed but did not always yield identifiable genetic isolation. These traits could be short‐lived or could be the origin of isolating reproductive lineages. Further interrogation of the genetic loci and gene expression pathways associated with complex courtship traits in luminescent ostracods will help improve our understanding of the speciation continuum.

## Author Contributions

**Nicholas J. Reda:** conceptualization (equal), data curation (equal), formal analysis (lead), investigation (equal), methodology (lead), software (equal), validation (equal), visualization (equal), writing – original draft (lead), writing – review and editing (supporting). **Vanessa L. González:** data curation (equal), formal analysis (equal), investigation (supporting), methodology (equal), software (equal), validation (equal), writing – review and editing (supporting). **Todd Osmundson:** data curation (supporting), formal analysis (supporting), methodology (supporting), writing – review and editing (supporting). **Gretchen A. Gerrish:** conceptualization (equal), data curation (equal), formal analysis (equal), funding acquisition (lead), investigation (equal), methodology (equal), project administration (lead), resources (equal), software (supporting), supervision (lead), validation (equal), visualization (equal), writing – original draft (supporting), writing – review and editing (lead).

## Conflicts of Interest

The authors declare no conflicts of interest.

## Data Availability

All physical and luminescent display data are submitted available on DRYAD https://doi.org/10.5061/dryad.kh18932m7. All sequences and read files are available on NCBI under BioProject ID: PRJNA1230213.
